# Longitudinal and cross-sectional sampling and whole genome sequencing of *Campylobacter* in a chicken abattoir reveal highly dynamic population structure

**DOI:** 10.1128/aem.02369-24

**Published:** 2025-05-09

**Authors:** Shanwei Tong, Kaidi Wang, Shenmiao Li, Michael Trimble, Yunxuan Chen, Lixue Liu, Jun Duan, Eduardo Taboada, Xiaonan Lu, William Hsiao

**Affiliations:** 1 Center for Infectious Disease Genomics and One Health, Faculty of Health Sciences, Simon Fraser University 241829, Burnaby, British Columbia, Canada; 2 Bioinformatics Graduate Program, The University of British Columbia 8166, Vancouver, British Columbia, Canada; 3 Food, Nutrition and Health Program, Faculty of Land and Food Systems, The University of British Columbia 117198, Vancouver, British Columbia, Canada; 4 Department of Food Science and Agricultural Chemistry, Faculty of Agricultural and Environmental Sciences, McGill University 151165 , Sainte-Anne-de-Bellevue, Québec, Canada; 5 Public Health Agency of Canada, National Microbiology Laboratory 85072 , Winnipeg, Manitoba, Canada; Anses, Maisons-Alfort Laboratory for Food Safety, Maisons-Alfort, France

**Keywords:** *Campylobacter*, poultry production, food safety, whole genome sequencing, comparative genomics

## Abstract

*Campylobacter* is a leading cause of human gastroenteritis worldwide and is commonly identified in poultry products. Current knowledge of its dissemination patterns in poultry production largely relies on the less sensitive traditional genotyping methods. In this study, whole-genome sequencing was applied to 324 *Campylobacter* isolates sampled from a chicken abattoir in the Greater Vancouver area throughout 2020. Core genome multi-locus sequence typing analysis revealed a highly diverse and dynamic *Campylobacter* population containing 27 distinct lineages. A wide range of plasmids was characterized, and a high prevalence of antibiotic resistance was observed among these isolates. Distinct subpopulations were identified in 10 lineages, suggesting that some *Campylobacter* populations may have diversified within the local agricultural environment. Some lineages were frequently reintroduced to the abattoir, suggesting the potential presence of hidden *Campylobacter* reservoirs upstream of slaughter. Comparisons between biological and environmental samples suggest a high probability of between-batch cross-contamination. Locally sourced public *Campylobacter* isolates showed strong genomic correlations with the lineages identified in this study. Notably, lineages 1629a and 1629b were identified to have persisted within the local poultry production ecosystem for several years, explaining their recurrent detection. In conclusion, this study enhances our understanding of *Campylobacter* population dynamics in the chicken abattoir environment, providing insights for controlling this foodborne pathogen in poultry production systems.

**IMPORTANCE:**

Using whole-genome sequencing, this study revealed a highly diverse and dynamic *Campylobacter* population within the chicken abattoir. The high prevalence of antibiotic resistance marked the critical need for surveillance in this region. The findings highlighted the likely existence of a hidden common source of *Campylobacter* upstream in the poultry production chain, which significantly contributes to the repeated introduction of the same lineages into the abattoir. Given the frequent reintroductions, the current understanding of *Campylobacter* persistence in the abattoir environment (up to 21 days) may require revision. Additionally, batch-to-batch dissemination of *Campylobacter* strains during processing is highly possible. A robust geographic association was also observed between the *Campylobacter* population in the abattoir and the local community. In sum, this study provides insights into the dynamics of *Campylobacter* contamination in the poultry production chain, offering guidance for improving prevention and control strategies.

## INTRODUCTION

*Campylobacter* is the leading cause of bacterial gastrointestinal enteritis globally (1). In Canada, campylobacteriosis is the most frequently reported bacterial foodborne illness, outnumbering the combined cases of *Listeria*, *Salmonella*, and *Escherichia coli* infections (2). Among species in the *Campylobacter* genus, *C. jejuni* and *C. coli* are responsible for the majority of human infections. Campylobacteriosis typically presents with symptoms such as bloody or watery diarrhea, fever, abdominal cramps, and vomiting. While most patients recover without medical intervention, approximately one-third of those affected receive antibiotic treatment, such as quinolones or macrolides (3). However, resistance to these antibiotics has been steadily increasing over the past few decades (4). In certain cases, *Campylobacter* infections can lead to severe complications, such as Guillain-Barré syndrome and/or irritable bowel syndrome. *Campylobacter* primarily resides in the gastrointestinal tract of endothermic animals, including both domesticated and wild species (5). Specifically, both *C. jejuni* and *C. coli* are prevalent inhabitants of the intestinal tracts in healthy livestock, domestic pets, and wild birds. Transmission of *Campylobacter* to humans occurs via various routes, and the most prevalent mode is the fecal-oral route. Human infections with *Campylobacter* arise from the consumption of inadequately cooked or contaminated food, particularly raw or undercooked poultry products (6).

The poultry meat production chain has been recognized as a reservoir for *Campylobacter* (7). Although the detection rate of *Campylobacter* in chicken hatcheries is nearly zero (8), a study showed that colonization could occur as early as a few days after hatching and rapidly spread to the entire flock (9). *Campylobacter* has been detected in various locations downstream of the poultry processing chain, including broiler farms, abattoirs, and retail stores. As the final step before broiler meat is packaged and delivered to markets, chicken abattoirs play a significant role in the batch-to-batch dissemination of foodborne pathogens carried by chickens. They are considered critical hubs for *Campylobacter* contamination within the poultry production chain (7, 10). A previous study found that while birds in a broiler barn were mostly colonized by a limited number of subtypes, the genomic diversity of *Campylobacter* increased significantly in the abattoir, suggesting that these facilities act as sites for cross-contamination (11). *Campylobacter* strains introduced by previous broiler batches could persist in the abattoir environment for over 21 days (12), leading to cross-contamination of subsequent batches of broiler meat (13). Additionally, machinery used in the abattoir contributes significantly to carcass contamination, with de-feathering and evisceration steps identified as hotspots for cross-contamination (14).

Over the past decades, studies of tracing pathogens in the poultry industry largely relied on the conventional typing techniques, such as pulsed-field gel electrophoresis and multi-locus sequence typing (MLST). Whole-genome sequencing (WGS) has emerged as the leading method for bacterial typing, offering notable advantages in these investigations (15). The increased subtyping resolution provided by WGS enables the identification of cases that might previously have been overlooked as sporadic (16). Unlike traditional methods that use only a limited set of genomic features to infer phylogeny, WGS enables precise pathogen tracking through high-resolution clustering at the whole-genome scale. Additionally, WGS empowers highly accurate predictions of phenotypes, such as pathogenicity and antibiotic resistance in *Campylobacter* (17). In recent years, the declining cost of WGS has confirmed it as the gold standard for tracking and surveillance of foodborne pathogens (18). Public health agencies such as the US Food and Drug Administration and Canadian Food Inspection Agency have applied WGS in routine foodborne pathogen surveillance to investigate potential outbreaks (19). As of November 2023, *Campylobacter* ranks as the third most frequently sequenced organism in the NCBI Pathogens database, surpassed only by *Salmonella* and *E. coli* (20).

Despite its frequent inclusion in government-led WGS surveillance programs, the application of WGS to *Campylobacter* in the context of food production remains underexplored. Currently available studies on the dissemination and persistence of *Campylobacter* in poultry production primarily rely on qualitative analyses or traditional typing methods, many of which suggested the critical role of chicken abattoirs as sources of cross-contamination (10, 11, 14, 21). The overall objective of this study is to characterize the *Campylobacter* population present in chicken abattoirs using WGS and to investigate its diversity and dynamics. To date, only one study has used WGS to examine *Campylobacter* populations in poultry processing plants (22). However, that study focused exclusively on biological samples from carcasses and meat. In contrast, the current study expands the scope to include environmental samples within the abattoir environment. Specifically, we aim to characterize the dissemination and persistence of *Campylobacter* populations in the abattoir using WGS.

## RESULTS

To investigate the diversity and dynamics of the *Campylobacter* population in the chicken abattoir, biological and environmental samples were collected weekly in a chicken abattoir located in the Greater Vancouver area in BC, Canada. Sampling was conducted in two rounds, each spanning 6 weeks during 2020 (Table 1). This study hypothesizes that upstream biological samples (i.e., chicken guts) represent the *Campylobacter* population colonizing the chicken flocks prior to slaughter. A total of 324 clones were confirmed as contamination-free *Campylobacter*, including 268 isolates of *C. jejuni* and 56 isolates of *C. coli*. The genomic diversity as well as the spatiotemporal distribution of these isolates was analyzed and is summarized below.

**TABLE 1:** Summary of sampling time points

Week number	Broiler farm ID	Sampling round	Week ID	Date of sampling	*Campylobacter* positivity rate^*a*^
1	1	Round 1	R1S1	2020-08-05	39.5%
2	2	Round 1	R1S2	2020-08-12	50.0%
3	3	Round 1	R1S3	2020-08-19	34.2%
4	4	Round 1	R1S4	2020-08-26	21.1%
5	5	Round 1	R1S5	2020-09-02	36.8%
6	6	Round 1	R1S6	2020-09-09	63.2%
7	7	Round 2	R2S1	2020-11-02	5.3%
8	8	Round 2	R2S2	2020-11-09	13.2%
9	9	Round 2	R2S3	2020-11-16	2.6%
10	2	Round 2	R2S4	2020-11-23	57.9%
11	10	Round 2	R2S5	2020-11-30	42.1%
12	11	Round 2	R2S6	2020-12-07	21.1%

^*a*^ Proportion of positive samples among all collected samples. Multiple *Campylobacter*-positive clones isolated from the same sample are counted as a single-positive sample.

### Genomic diversity and profile characterization of *Campylobacter* isolates

**Fig 1:**
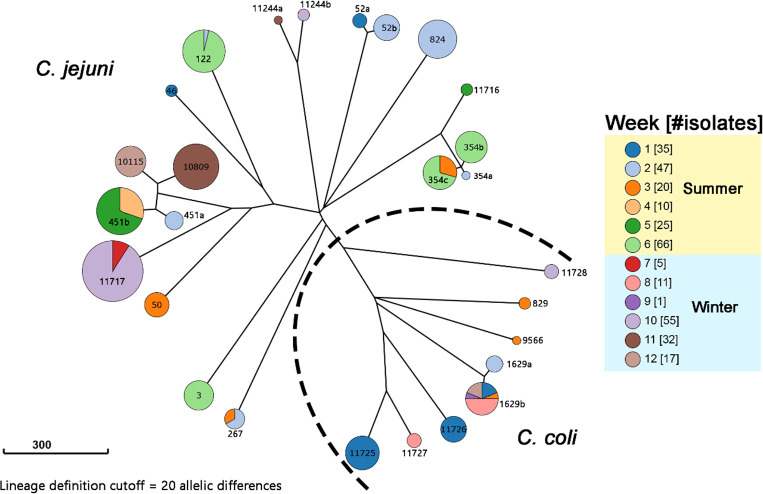
The cgMLST neighbor-joining tree of all isolated *Campylobacter* strains. Each cluster represents a lineage, with the lineage names labeled on the clusters. Lineages are named based on their ST number, with additional letters indicating multiple clusters identified within the same ST. A clustering threshold of 20 allelic differences was applied. The scale bar on the left represents a branch length of 300 allelic differences. Cluster colors indicate the isolation time of each lineage, and the total number of isolates collected each week is shown in the legend. A dotted line separates *C. jejuni* isolates from *C. coli* isolates.

Using *in silico* MLST analysis, 22 sequence types (STs) were determined among the *Campylobacter* isolates. A total of seven novel STs were identified, including ST-11716 from the ST-324 clonal complex (CC), ST-11717 from the ST-21 CC, ST-11725, ST-11726, and ST-11727 from the ST-828 CC, ST-11728 from the ST-1150 CC, and ST-11729 as a singleton (unassigned to any CC in PubMLST). Core genome MLST (cgMLST) phylogenetic analysis revealed clustering within the same STs, resulting in the definition of 27 *Campylobacter* lineages based on a cgMLST clustering threshold of 20 allelic differences (Fig. 1; Table S1). Each lineage was named according to its ST, with additional letters (i.e., “a”, “b”, “c”) appended to differentiate multiple lineages within the same ST.

ST-451 isolates consisted of two lineages, with one (451a) identified in week 2 and the other (451b) in weeks 4 and 5. ST-354 isolates contained three lineages: 354a (week 2), 354b (week 6), and 354c (weeks 3 and 6). ST-52 isolates were composed of two lineages, with lineage 52a identified in week 1 and lineage 52b identified in week 2. ST-1629 isolates comprised two lineages, with 1629a identified in week 2 and 1629b identified spanning weeks 1, 3, 8, 9, and 12. Notably, ST-11729 (one isolate, week 10) and ST-11717 (54 isolates, weeks 7 and 10) isolates belonged to the same lineage 11717. A single point mutation at the 905th position of the *aspA* housekeeping gene resulted in the shift of ST, although this mutation was insufficient to differentiate ST-11729 from ST-11717 at the strain level.

*C. jejuni* was detected in all sampling weeks except weeks 8 and 9, while *C. coli* was identified in weeks 1, 2, 3, 8, 9, 10, and 12. *C. coli* isolates outnumbered *C. jejuni* isolates only in weeks 1, 8, and 9. Low isolate numbers (<10) were obtained in weeks 7 and 9, with only one *Campylobacter* isolate recovered in week 9. The remaining isolates from this week were subsequently identified as *Arcobacter* and excluded from the study.

**Fig 2:**
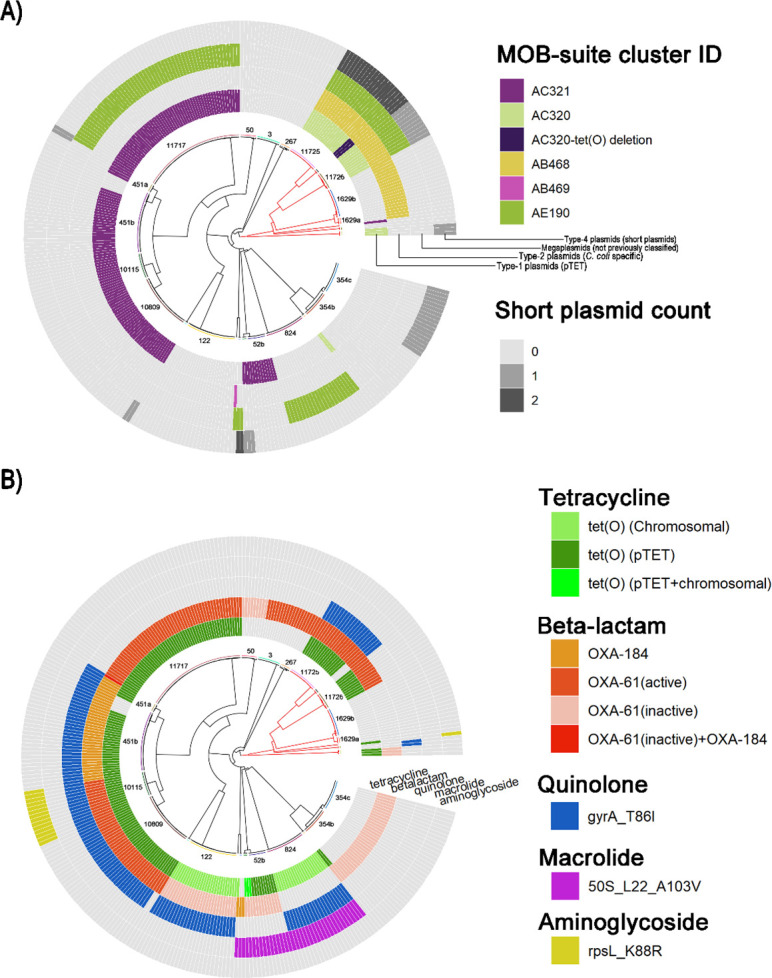
Plasmid and antimicrobial resistance (AMR) profiles of all *Campylobacter* isolates. *C. jejuni* lineages are shown in black and *C. coli* lineages are shown in red. STs are labeled on the respective lineages. (**A**) Plasmid profiles of all 324 isolates. Large plasmids (>10 kb), including type 1, type 2, and megaplasmids, were classified into distinct MOB-suite clusters indicated by different colors. Smaller plasmids (<6 kb, type 4) were grouped and colored based on count. (**B**) Genetic elements associated with different types of AMR identified in the isolates. These include genes conferring resistance to beta-lactamases and tetracycline, as well as point mutations linked to resistance against quinolones, macrolides, and aminoglycosides.

A total of 448 plasmids were identified and characterized among all *Campylobacter* isolates (Fig. 2A). These plasmids were classified into 10 primary clusters using MOB-suite (23, 24), with 9 of the clusters manually categorized into either type-1 (tetracycline resistance-containing plasmid, also known as pTet), type-2 (*C. coli-*specific plasmids), type-3 (also known as pVir), or type-4 (short plasmids with limited sequence similarity) plasmids (25). Clusters AC320 and AC321 were assigned to type-1 plasmids. Interestingly, lineage 11727 exhibited a deletion of *tet(O*) within type-1 plasmids. However, these plasmids retained other pTet-like features, such as the presence of a *virB*-type IV secretion system. In lineage 451b, six isolates lacked AC321, forming a distinct subpopulation within this lineage (discussed further below). Type-2 plasmids are approximately 30 kb in length and have a type IV secretion system as well as *virB* genes. While cluster AB468 is unique to *C. coli*, cluster AB469 was found in a *C. jejuni* isolate, which was not previously reported. Five clusters, namely AC501, AC502, AC439, AC498, and AD894, were shorter than 6 kb, lacked strong sequence similarity, and were assigned to type-4 plasmids. Identified by MOB-suite, cluster AE190 could not be assigned under any of the aforementioned types. Plasmids with features similar to AE190 were recently reported as megaplasmids (6, 26). The AE190 megaplasmids are over 100 kb in size and encode a clinically significant type VI secretion system (27) along with a suite of conjugation transfer *Tra* and *Trb* proteins.

AMR was commonly predicted across the lineages (Fig. 2B), with resistance to five distinct classes of antimicrobials identified. Beta-lactam resistance genes were detected in 23 lineages, representing 86% of the isolates. These genes were found in the form of either the *OXA-61*-like gene (*Cj0299*) or the OXA-184 gene. The transcriptional activity of *OXA-61*-like genes in *Campylobacter* is regulated by a G→T transversion in the promoter region, located 57 bp upstream of the start codon (28). Approximately 63% of the *OXA-61*-like genes were predicted to exhibit high expression levels due to this mutation. Five variants of *OXA-61*-like genes were identified, namely *OXA-61*, *OXA-193*, *OXA-460*, *OXA-594,* and *OXA-603*. The tetracycline resistance gene *tet(O*) was identified in 19 lineages. This gene was localized either on the chromosome (22%), on pTet plasmids (77%), or on both (1%). Notably, one clade of ST-52 (three isolates) showed two non-homologous copies of *tet*(O), with one on the chromosome and the other on the pTet plasmid. The most frequently identified AMR types among the isolates were resistance to tetracycline (71%), beta-lactams (59%), and quinolones (48%). Isolates within the same lineage typically exhibited consistent plasmid AMR profiles. An exception was observed in lineage 451b, where a subset of isolates lacked the pTet plasmid and associated tetracycline resistance. Multi-drug resistance profiles were commonly predicted among the *Campylobacter* isolates. Lineage 10115 demonstrated resistance to four distinct antibiotic classes, while six other lineages (451a, 451b, 10809, 824, 11244, and 11725) exhibited resistance to at least three classes.

### Population structure within respective lineages

**Fig 3:**
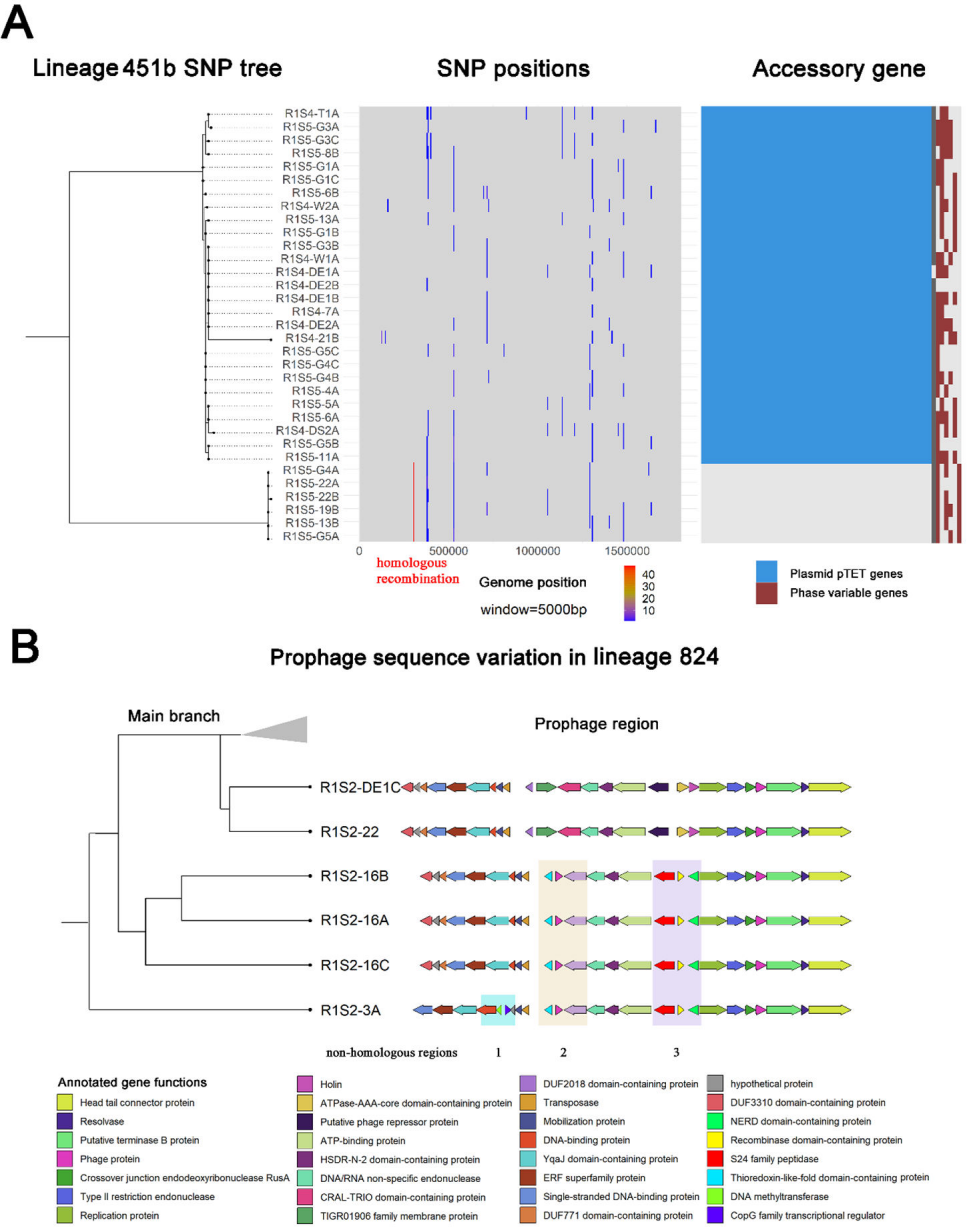
Population structures within individual clusters. (**A**) Population structure of lineage 451b defined by variations in recombination and plasmid profiles. The SNP phylogeny is shown in the left panel, while the middle panel shows SNP positions relative to the reference strain *C. jejuni* NCTC-11168, plotted in their genomic locations using a 5,000 bp window. A homologous recombination region with elevated SNP density (highlighted in red) was identified among the six bottom isolates. The right panel displays the accessory gene profile, indicating that a tetracycline resistance plasmid (pTET) is present only in the main branch and absent in these six isolates. Phase-variable genes do not show significant differences between the two populations. (**B**) Population structure of lineage 824 defined by variations in prophage profiles. The SNP phylogeny is shown alongside gene annotations of a prophage region. A portion of the main branch is collapsed, with all isolates in the main branch sharing the same prophage region as the top 2 isolates shown in this figure. Isolates R1S2-16A/B/C exhibit differences in the prophage region in non-homologous regions 2 and 3, while isolate R1S2-3A shows differences in regions 1, 2, and 3 compared to the main branch.

Distinct population structures were identified in 10 out of the 27 identified lineages. Horizontal gene transfer and/or point mutations contributed to the formation of subpopulations within these isolates. Recombination defined the subpopulations of lineages 267, 451b, 1629b and 11726. For example, lineage 451b consists of 10 week 4 isolates and 22 week 5 isolates. Within this group, six week 5 isolates were distinguished by a chromosomal homologous recombination region and the loss of the pTet plasmid (Fig. 3A). The recombination region is 2.6 kb in length and contained 52 substitutions and one insertion in a highly condensed region encompassing genes *Cj1062–Cj1068*, which are involved in the metabolic process. In this region, the sequence in the main clade in lineage 451b was identical to that of lineage 451a, while differences were observed exclusively in the 451b subpopulation. This demonstrates a higher probability that the subpopulation diverged from the main clade than *vice versa*. The subpopulation was isolated from both gut and machine samples, and some samples (e.g., R1S5-G4) contained strains from both the main clade and the subpopulation, suggesting that the subpopulation likely diverged and co-existed with the main clade within the same broiler flock before arriving at the abattoir.

Variation in prophage sequences defined the subpopulations in lineages 3, 50, 824, and 11725. In each case, the prophage sequences underwent modifications, resulting in different variants. In lineage 824 isolates, variations in a prophage region were observed in four isolates (Fig. 3B). Isolate R1S2-3A exhibited a distinct variant compared to R1S2-16A/B/C, containing a DNA-binding protein gene and several unique genes, such as DNA methyltransferase and a transcriptional regulator. Both variants showed differences in homologous and non-homologous regions compared to the main clade. In contrast, the subpopulation in lineages 1629b and 11717 was defined by point mutations. In lineage 11717 (Fig. S1), 10 point mutations differentiated week 7 isolates from week 10 isolates. These single nucleotide polymorphisms (SNPs) were scattered across various genomic regions. This contrasts with the subpopulation in lineage 451b, where SNPs were concentrated in a single region due to a single horizontal gene transfer event. Lineages 1629b and 11717 were the only lineages where subpopulations were defined by point mutations and where population structure could be distinctly separated based on the isolation date.

### Time and space distribution of isolated *Campylobacter* lineages

**Fig 4:**
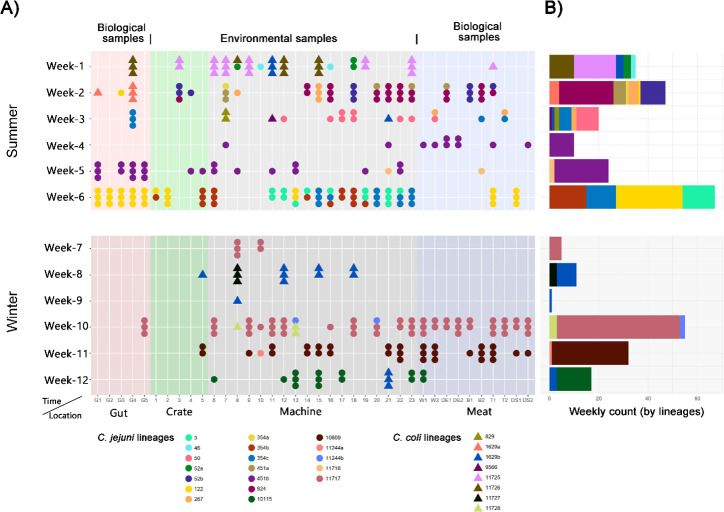
Temporal and spatial distributions of the detected *Campylobacter* lineages. (**A**) Distribution map of *C. jejuni* and *C. coli* lineages across different sampling locations and dates. Each lineage is represented by a distinct color and labeled with its corresponding sequence type number. Clones derived from the same sample are stacked together. (**B**) The frequency of each lineage detected at various sampling time points.

The temporal and spatial distribution as well as the genomic diversity of all isolated *Campylobacter* clones are shown in Fig. 4. Machine samples tested positive for *Campylobacter* on all 12 sampling dates, recording the highest positive rate (41.6%) among all sampling sections. The positive rates for machine samples collected in the evisceration room and the “Whole bird lane, after cooling” section were 40.0% and 47.2%, respectively. After chicken carcasses were portioned, the positive rates in the respective processing lanes were as follows: 37.5% for the winglet lane, 37.5% for the drumette lane, 50.0% for the back-on-breast (BOB) lane, and 41.6% for the thigh and leg lane. Meat samples exhibited an overall positive rate of 28.3%, including 33.3% in winglet, 20.8% in drumette, 29.2% in BOB, 33.3% in thigh, and 25.0% in drumstick samples. Besides, 25.0% of gut samples and 16.7% of crate samples tested positive. From round 1 (summer) to round 2 (winter), significant reductions in positivity rates were observed for both gut and crate samples, dropping from 46.7% to 3.3% and 22.2% to 6.7%, respectively. A detailed summary of positivity rates by location is provided in Table 2.

**TABLE 2:** Summary of sampling locations

Location	Sample	Sampling method	Number of samples taken	Sample IDs	*Campylobacter* positivity rate
Live end	Transporting crates	Swab	5	1, 2, 3, 4, 5	16.7%
Evisceration room	Evisceration room venter	Swab	1	6	41.6%
Evisceration room	Carcass opener	Swab	1	7	33.3%
Evisceration room	Eviscerator	Swab	1	8	58.3%
Evisceration room	Eviscerated chicken gut	Organs	5	G1, G2, G3, G4, G5	25.0%
Evisceration room	Cropper	Swab	1	9	25.0%
Evisceration room	Lung sucker	Swab	1	10	33.3%
Whole bird lane, after cooling	Rehanger	Swab	1	11	50.0%
Whole bird lane, after cooling	Transfer	Swab	1	12	50.0%
Whole bird lane, after cooling	Wing stretcher	Swab	1	13	33.3%
Winglet lane	Wing chute	Swab	1	14	25.0%
Winglet lane	Wing conveyor	Swab	1	15	50.0%
Winglet lane	Wing meat	Rinse	2	W1, W2	33.3%
Drumette lane	Drumette chute	Swab	1	16	50.0%
Drumette lane	Drumette conveyor	Swab	1	17	25.0%
Drumette lane	Drumette meat	Rinse	2	DE1, DE2	20.8%
BOB lane	BOB chute	Swab	1	18	50.0%
BOB lane	BOB meat	Rinse	2	B1, B2	29.2%
Thigh/Leg lane	Bypass	Swab	1	19	25.0%
Thigh/Leg lane	JLR	Swab	1	20	25.0%
Thigh/Leg lane	Drum cutter	Swab	1	21	58.3%
Thigh/Leg lane	Thigh conveyor	Swab	1	22	50.0%
Thigh/Leg lane	Thigh meat	Rinse	2	T1, T2	33.3%
Thigh/Leg lane	Drum conveyor	Swab	1	23	50.0%
Thigh/Leg lane	Drumstick meat	Rinse	2	DS1, DS2	25.0%

Distinct lineages were occasionally identified from different clones of the same sample, and co-occurrence of multiple lineages at a single time point was common. Weeks 1–3 showed high lineage diversity, each containing five or more lineages, while weeks 4, 7, and 9 contained isolates from only a single lineage. Some lineages reappeared at different sampling dates throughout the study. Most lineages were identified at most twice, typically within the same round (summer or winter), including lineages 122, 267, 354c, 451b, 11717 (Fig. 1 and 4). The only exception was lineage 1629b, which appeared at five different time points at weeks 1, 3, 8, 9, and 12. Among the recurring lineages, six (122, 267, 354c, 451b, 1629b, and 11717) were identified across multiple time points, with evidence suggesting reintroduction to the abattoir by different broiler batches. For example, lineage 451b was prevalent in week 4 samples and reappeared in week 5. Its presence in gut samples during week 5 indicated that the broiler flock processed that week carried lineage 451b before slaughter rather than facility contamination from week 4. Similarly, lineage 354c was isolated from one gut sample and two meat samples in week 3 and later detected in two crate samples and six machine samples in week 6. Given that *Campylobacter* cannot proliferate in aerobic environments, such as machine surfaces (29), persistence does not explain the observed increase in positive samples or the absence of lineage 354c in weeks 4 and 5. These strongly suggest reintroduction by broiler chickens as the primary driver of recurring lineages, with no apparent evidence supporting environmental persistence within the abattoir.

Our observations strongly suggest dissemination between biological and environmental samples. Each week, at least one environmental sample carried a lineage not identified in gut samples from the same week, suggesting these lineages were likely carried over from previous broiler batches. Conversely, gut lineages were found in environmental samples during weeks 1, 5, 6, and 10, suggesting the dissemination of *Campylobacter* from broiler chickens to machinery. In nine time points (weeks 1–6, 10–12) where biological samples (gut or meat) tested positive for *Campylobacter*, at least one lineage was shared with environmental samples from the same week, indicating the likelihood of cross-contamination. Meat samples tested positive while gut samples were negative in weeks 4, 11, and 12. Moreover, during weeks 1, 2, 3, and 5, meat samples shared lineages with environmental samples that were absent in gut samples. These findings indicated the role of the abattoir environment as a platform for *Campylobacter* cross-contamination and batch-to-batch dissemination.

### Geographical distribution of public isolates associated with lineages in this study

**Fig 5:**
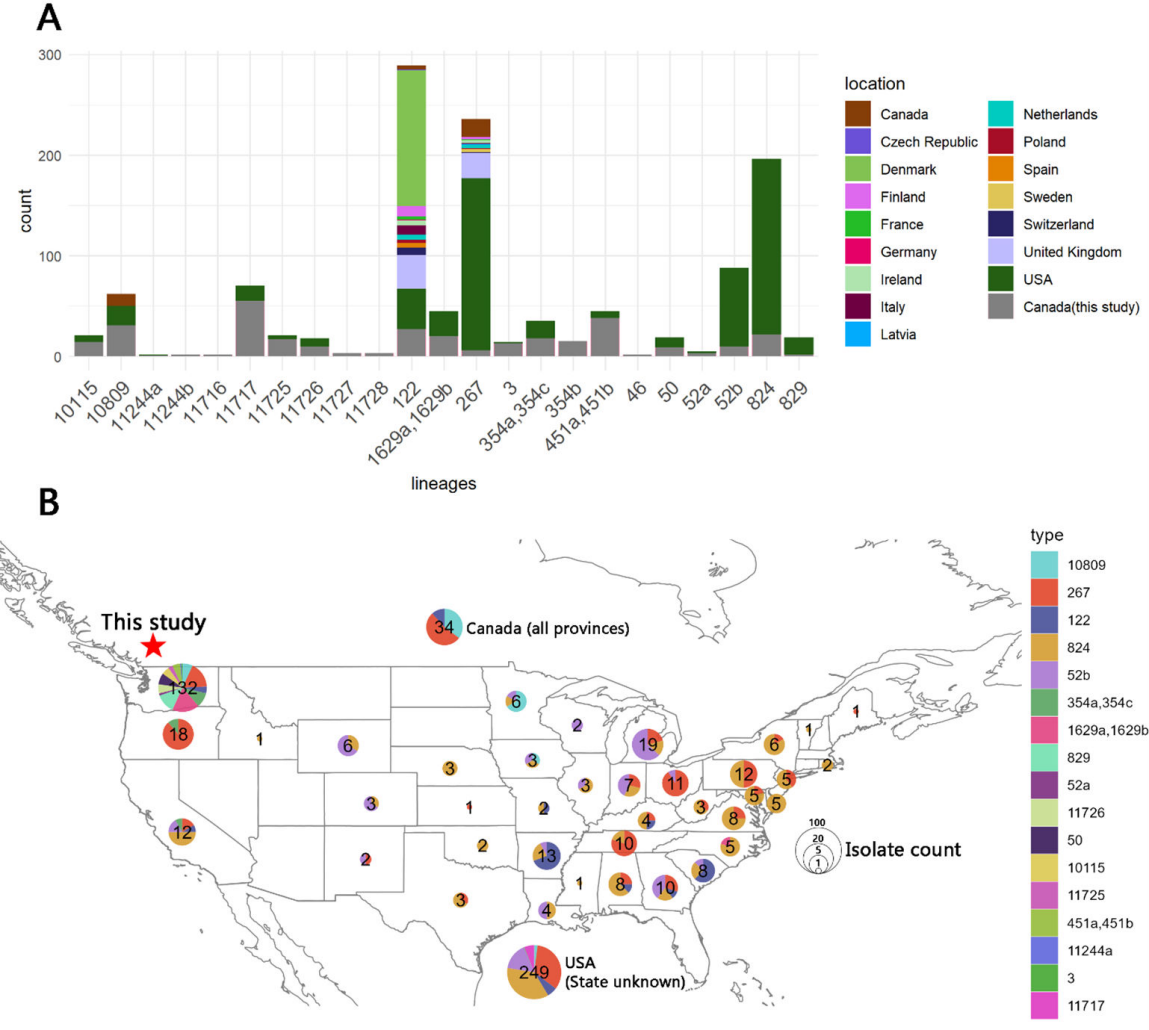
Geographic distribution of public strains associated with lineages identified in this study. (**A**) Bar plot showing the lineages defined in this study (gray) alongside associated public strains (colored by their geographic location of isolation). Closely related lineages (e.g., 1629a and 1629b) belonging to the same SNP cluster were merged into a single column. (**B**) Geographic distribution of all North American public isolates. US isolates were further categorized by state. The isolate count and genomic diversity in each state or region are depicted using pie charts. The size of each pie chart along with the number displayed in its center represents the isolate count. Genomic diversity is indicated by the colors of the chart, corresponding to the associated lineages. The map was visualized using the maps R package.

To explore the global association of the lineages identified in this study, we analyzed all available *Campylobacter* strains in the NCBI Pathogens database (20) as of November 2023. Among the 27 lineages identified, 20 (corresponding to 17 SNP clusters) had associated isolates in the database (Table 3). In three cases, pairs of lineages (1629a + 1629b, 354a + 354c, and 451a + 451b) were assigned to the same SNP cluster. A total of 1,000 public isolates were linked to lineages from this study (Table S2). A strong geographic signal was observed in most of the associated SNP clusters, with their global distribution depicted in Fig. 5A. The majority of associated public isolates were from the USA. Isolates linked to lineages 122 and 267 included strains from outside North America, some of which were collected in European countries. Figure 5B highlights the regional distribution of US isolates by state. Washington state accounted for 132 associated isolates, while no other state exceeded 20 isolates. The Washington isolates were highly diverse, corresponding to 14 SNP clusters (associated with 17 lineages), which represented 13% of all Washington isolates in the database (Table S3). In contrast, isolates from other states, particularly in the central and eastern regions, exhibited limited diversity. SNP clusters PDS000022406.165 (associated with lineage 267), PDS000021354.132 (associated with lineage 824), and PDS000011725.73 (associated with lineage 52b) predominated in these states. Interestingly, isolates associated with lineages 824 and 52b were absent in Washington, suggesting that these lineages may not have been introduced locally.

**TABLE 3:** Association between the lineages identified in this study and SNP clusters from the NCBI Pathogens database

NCBI Pathogen SNP cluster^*a*^^,^^*b*^	Lineage(s) in this study	Species
PDS000157656.1	3	*C. jejuni*
PDS000066025.10	50	*C. jejuni*
PDS000038425.2	52a	*C. jejuni*
PDS000011725.73	52b	*C. jejuni*
PDS000021225.73	122	*C. jejuni*
PDS000022406.165	267	*C. jejuni*
PDS000021101.12	354a, 354c	*C. jejuni*
PDS000153419.2	451a, 451b	*C. jejuni*
PDS000021354.132	824	*C. jejuni*
PDS000076138.3	10115	*C. jejuni*
PDS000022661.30	10809	*C. jejuni*
PDS000157645.1	11244a	*C. jejuni*
PDS000021215.13	11717	*C. jejuni*
PDS000021617.17	829	*C. coli*
PDS000038424.26	1629a, 1629b	*C. coli*
PDS000097386.4	11725	*C. coli*
PDS000060346.6	11726	*C. coli*

^*a*^ SNP cluster IDs from the NCBI Pathogens database are subject to change as new isolates are added. The IDs referenced in this study were retrieved on 13 November 2023.

^*b*^ Only SNP clusters containing both public isolates and isolates from this study are included. Among 27 lineages identified in this study, 26 were assigned to the corresponding SNP clusters. Isolate R1S3-11A, the sole representative of lineage 9566, was categorized as a singleton. Six lineages (46, 354b, 11716, 11244b, 11728, and 11727) were assigned to respective novel SNP clusters that are not listed.

**Fig 6:**
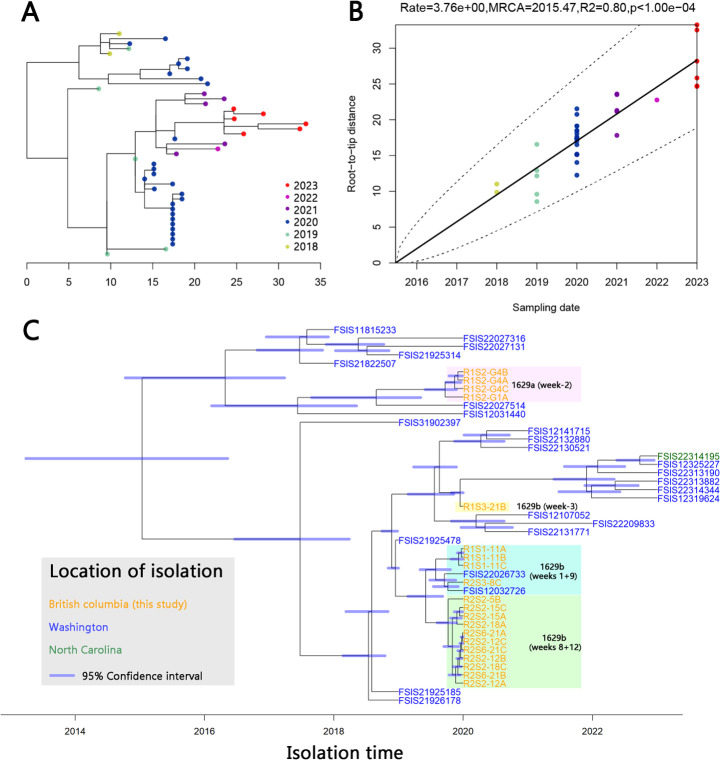
Time-based phylogenetic analysis of lineage 1629a and 1629b isolates along with associated public isolates. (**A**) Rooted core-genome SNP maximum-likelihood tree. The color of each node indicates the isolation time of the respective isolates. (**B**) Root-to-tip distance regression plot, showing a strong correlation between branch distances and sampling time (*R* = 0.80). (**C**) Time-based phylogenetic tree constructed using BactDating. Blue horizontal bars at each internal node indicate the 95% confidence intervals of the estimated coalescence times. The location of isolation is annotated using text color, and isolates from this study are highlighted. Lineages 1629a and 1629b are located on separate branches, with their most recent common ancestor estimated to date back to 2013–2016. The lineage 1629b isolates from this study share a common ancestor with numerous public isolates located on the bottom branch.

The recurring presence of lineage 1629b throughout the year (Fig. 1 and 4) suggests that this *C. coli* lineage is particularly prevalent within the local poultry processing chain. To test this hypothesis, we analyzed all 25 isolates from the same SNP cluster (PDS000038424.26) as lineages 1629a and 1629b were downloaded from the NCBI Pathogens database (Table S4). These isolates were collected in the USA by FSIS-USDA between 2018 and 2023, with 24 from Washington and 1 from North Carolina. This SNP cluster was also the largest identified in Washington (Table S3). Similar to the isolates from this study, all public isolates were collected within the poultry production chain primarily from broiler environments or broiler meat. A maximum-likelihood SNP phylogeny of lineage 1629a and 1629b isolates in this study, as well as associated public isolates, was constructed after removing recombination in the sequence alignment (Fig. 6A). Time-based integration revealed a strong correlation between root-to-tip distances and collection dates (Fig. 6B). The resulting time-based phylogenetic tree (Fig. 6C) demonstrated significant divergence within lineage 1629b isolates across weeks 1 + 9, week 3, and weeks 8 + 12. Lineage 1629b and a subset of Washington public isolates appeared to have diverged from a common ancestor around 2019. This finding supports the hypothesis that the lineage diversified within the local agroecosystem over several years and was repeatedly introduced to the abattoir. Moreover, lineages 1629a and 1629b were positioned on separate branches, descending from a common ancestor estimated to have existed between 2013 and 2016. These findings suggest that lineages 1629a and 1629b have become established in the poultry production ecosystem of the Pacific North Coast region. The time-based phylogeny estimates that colonization of this SNP cluster may have started approximately 10 years ago.

## DISCUSSION

This study employed a combination of longitudinal and cross-sectional sampling to trace and characterize the *Campylobacter* population in a chicken abattoir. Weekly sampling was conducted over 12 weeks, covering various stages from the entry of broiler chickens to the final meat products. The overall prevalence of *Campylobacter* in the abattoir was 38.2%, slightly lower than reported in some previous studies (22, 30). A higher prevalence of *Campylobacter* was observed during the summer round (43.0%) compared to the winter round (33.3%). The summer round also consisted of greater lineage diversity, with 20 lineages identified, compared to eight lineages in the winter round. These findings align with the thermophilic nature of *Campylobacter* and are consistent with prior research highlighting the seasonality of this pathogen within the poultry production chain (4, 31). This study unveiled a remarkably diverse *Campylobacter* population in the abattoir, with predominant lineages undergoing continuous and rapid shifts over time.

By characterizing the genomic profiles of the isolates, this study identified a variety of plasmids and observed a high prevalence of AMR. Notably, an uncommon observation of a *C. coli*-specific type-2 plasmid in *C. jejuni* was reported along with a *tet(O*)-deleted pTet variant. AMR was highly prevalent, with 80% of isolates carrying at least one identified resistance marker. Alarmingly, a similarly high AMR prevalence among *Campylobacter* isolates from poultry products in the Greater Vancouver region was reported in a previous study (32), highlighting the critical need for continued AMR surveillance in the local poultry industry. Previous studies have demonstrated that WGS-based predictions of *Campylobacter* AMR exhibit strong concordance with traditional antimicrobial susceptibility tests (17, 33). These predictions effectively identify resistance through mechanisms such as gene presence (e.g., tetracycline resistance), point mutations (e.g., quinolone resistance), and transcription activity (beta-lactamase production). Government-led WGS-based AMR surveillance programs, such as the National Antimicrobial Resistance Monitoring System in the USA, have already been implemented to enhance food safety (17, 34).

The frequent presence of subpopulations within lineages may reflect the adaptation of *Campylobacter* to the agricultural environment. Recognized for its high recombination rates due to natural competence (35), *Campylobacter* relies on horizontal gene transfer as a key mechanism in driving genetic diversification and defining subpopulations within eight lineages. Notably, the subpopulation in lineage 451b was associated with the loss of a tetracycline resistance plasmid, potentially indicating a survival cost for maintaining pTet under Canadian broiler farm conditions (36). This observation aligns with the strict prohibition of preventive tetracycline use in Canadian poultry farms since 2018 (37). To better understand the evolutionary implications of horizontal gene transfer within these lineages, further investigation is needed to compare the fitness differences between the main clade and its subpopulation.

We hypothesize that *Campylobacter* lineages colonize and diversify within the local poultry breeding environment and are repeatedly transported to the abattoir from different sources. A notable example is lineage 122 observed in weeks 2 and 6, which was identified in gut samples from broiler batches raised on different farms (Table 1). We initially expected each reintroduction to correspond to a distinct subpopulation. However, in four out of six reintroduced lineages, including lineage 122, even highly sensitive WGS failed to identify genomic variations between different introductions, suggesting minimal divergence time among these recurrences. In contrast, lineages 1629b and 11717 exhibited distinct subpopulations at each time point defined by point mutations, indicating noticeable divergence over time. The time-based phylogeny demonstrated that subpopulations of lineage 1629b diverged from a common ancestor approximately 1 year ago, while lineages 1629a and 1629b diverged between 2013 and 2016 (Fig. 6). These findings suggest the potential existence of a hidden reservoir, such as water or broiler feed, acting as a common source of contamination. This reservoir could disseminate *Campylobacter* to multiple poultry farms in the region, contributing to the recurrent introductions observed in this study.

The constant reintroduction of local *Campylobacter* strains complicates the direct observation of bacterial persistence in the abattoir environment. Since there is no clear evidence that subsequent populations are direct descendants of the prior populations, it is challenging to make reliable inferences about *Campylobacter* persistence in this setting. A previous study estimated that *Campylobacter* could persist in an abattoir environment for up to 21 days based on traditional subtyping, assuming that recurring genotypes represented persistent strains (12). Using the highly sensitive WGS data, we contend that this duration should be re-evaluated due to the frequent reintroduction of strains. In addition to the impact of local strain reintroductions, differences in sanitization protocols among processing plants may significantly affect bacterial persistence, potentially contributing to discrepancies between studies. To accurately determine the actual duration of *Campylobacter* persistence, a revised sampling plan is necessary. Ideally, this plan would involve controlling the incoming broiler batches, conducting sampling as frequently as possible, and evaluating persistence under different sanitization strategies.

The same clones of *Campylobacter* are frequently seen between biological and environmental samples within the chicken abattoir, suggesting a significant likelihood of batch-to-batch contamination. However, this could not be directly tested in the current study due to the week-long intervals between sampling time points. Previous WGS-based investigations into *Campylobacter* contamination in the poultry industry have predominantly focused on biological samples, such as broiler carcasses (22, 38). For example, one study compared lineages from carcass and caeca samples and concluded that the main source of *Campylobacter* contamination in meat products was not the cross-contamination from previous batches in the abattoir environment but rather the bacterial population inherently carried by the broilers (22). In contrast, this study indicated that extensive environmental sampling alongside biological sampling can offer a more comprehensive understanding of *Campylobacter* cross-contamination patterns in the abattoir. By integrating WGS and environmental sampling, we determined that the abattoir environment may act as a hub for cross-contamination, where *Campylobacter* isolates are frequently acquired from chicken flocks and subsequently disseminated to broiler meat.

A strong geographical association was observed between the population of *Campylobacter* in the abattoir and publicly available data. Although only a few public strains were isolated in British Columbia, the proximity of the abattoir to the US-Canada border enabled comparisons with a well-sampled *Campylobacter* population from the neighboring state of Washington in the USA (Table S3). The Washington population closely mirrored the diversity observed in the abattoir, with 74% of the lineages being associated with isolates from Washington. Interestingly, certain lineages such as 824 and 52b were not present in the Washington population but were speculated to have been acquired from more distant sources. Phylogenetic analysis of both local and public isolates revealed the current dominance of the highly prevalent SNP cluster PDS000038424.26 (associated with lineages 1629a and 1629b) within the broiler production chain in Washington. These findings suggest that this SNP cluster is well adapted to the local poultry production ecosystem, potentially explaining its frequent, year-round reintroduction, while playing a significant role in shaping the *Campylobacter* population observed in the studied abattoir.

In conclusion, this study revealed a diverse and dynamic *Campylobacter* population within a chicken abattoir through comprehensive longitudinal and cross-sectional sampling. The high prevalence of AMR highlighted the critical need for ongoing AMR surveillance in the region. Subpopulations within *Campylobacter* lineages were identified, indicating that some lineages diversified either within the same broiler batch or across different batches. Further investigation is needed to understand the potential survival advantages conferred by the mutations defining these subpopulations. Dissemination of *Campylobacter* from carcass to machinery and from machinery to meat was found to be highly common, emphasizing the need for advanced poultry processing technologies to reduce between-batch cross-contamination. The duration of *Campylobacter* persistence in the abattoir should be reassessed, and optimal sanitization strategies should be developed to minimize bacterial persistence. This study emphasized the significant role of locally colonized lineages in shaping the *Campylobacter* population in the abattoir. The potential sources of these lineages are diverse. Some geographically adapted lineages may have originated locally, while others could have been introduced from external regions. Further studies are needed to investigate possible hidden reservoirs upstream in the poultry production chain, such as farms, transportation, chicken feed, and water sources.

## MATERIALS AND METHODS

**Fig 7:**
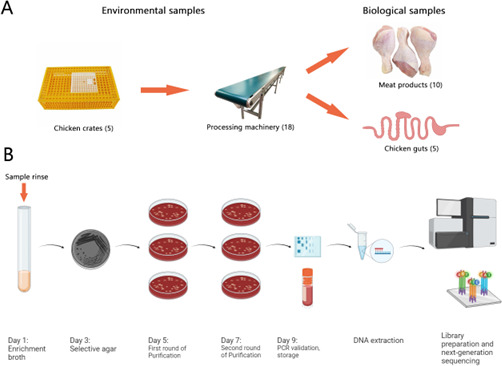
Schematic illustration of sample collection and processing procedures used in this study. This figure was created using Biorender.com. (**A**) Graphic summary of sampling locations and the corresponding number of samples collected. This includes environmental samples, such as swabs from chicken transportation crates and processing machinery, as well as biological samples including chicken guts and meat products. (**B**) Graphic summary of sample processing procedures.

The sampling schedule and locations are summarized in Tables 1 and 2. A schematic illustration of the sample collection and processing workflow is shown in Fig. 7.

### Collection of samples from chicken abattoir

All samples were collected from a chicken abattoir located in the Greater Vancouver region in BC, Canada. The abattoir operates with a two-shift system, comprising a morning and an afternoon shift. During each shift, the abattoir processes one flock of broiler chickens received from a poultry farm. Sampling was conducted weekly over 12 weeks, divided into two rounds: 6 consecutive weeks from August to September 2020 (round 1) and 6 consecutive weeks from November to December 2020 (round 2). On each sampling day, a single batch of broiler chickens was monitored from entry into the abattoir through slaughter and production of final products. The broiler batches tracked in weeks 2 and 10 originated from the same farm, while all other batches were sourced from different farms (Table 1).

Each week, 38 samples were collected from four sections of the abattoir, including two types of environmental samples (swabs from chicken crates and processing machinery) and two types of biological samples (chicken guts and meat product rinses). A total of 456 samples were collected over the 12 week study period. Gut samples were obtained in the evisceration room by randomly selecting and collecting different organs of the chicken, such as intestine, gizzard, and liver, subsequent to the evisceration process. On each sampling day, five different gut parts were collected for analysis. Crate samples were obtained by swabbing the crates used for transporting the flock of chickens. Five crate samples were collected on each sampling day. Machinery samples were acquired by swabbing the surface of various machineries situated throughout the processing line. Eighteen machine swabs were collected on each sampling day. For all swabbing procedures, a sampling sponge pre-soaked in buffered peptone water was employed (3M, London, ON, Canada). Meat samples were taken by rinsing broiler meat from five different parts, namely winglet, drumette, BOB, thigh, and drumstick, using buffered peptone water. For each chicken part, 4 pounds of meat were rinsed with 800 mL of buffered peptone water (3M, London, ON, Canada), and then two samples (10 mL each) were taken from the rinse.

The collected gut parts, sampling sponges, and meat rinse were placed into separate sterile sampling bags, with each containing 25 mL of buffered peptone water. The sampling bags were homogenized by gently massaging for 5 minutes.

### Enrichment, selection verification, and naming of *Campylobacter* isolates

After homogenization, 0.5 mL of the homogenized liquid was collected and enriched in 10 mL of Bolton broth (Sigma-Aldrich, Oakville, ON, Canada) supplemented with antibiotics (20 mg/L cefoperazone, 20 mg/L vancomycin, 20 mg/L trimethoprim, and 10 mg/L amphotericin B) at 37°C under microaerobic condition (10% CO_2_) for 48 h. In this step, modest concentrations of antibiotics were added to inhibit the growth of competing microbes while minimizing cellular damage. The bacterial culture was then inoculated onto modified charcoal cefoperazone deoxycholate agar (Sigma-Aldrich, Oakville, ON, Canada) plates supplemented with antibiotics (10 mg/L rifampicin, 7 mg/L polymycin B, 10 mg/L trimethoprim, 10 mg/L cycloheximide, 128 mg/L cefoperazone, and 4 mg/L amphotericin B) at 37°C under microaerobic conditions for an additional 48 h. In this step, higher concentrations of antibiotics were added to eliminate residual background flora. We selected 37°C instead of the standard 42°C typically used for *C. jejuni* and *C. coli*, to facilitate the detection of emerging *Campylobacter* species such as *C. fetus*, which grows at 37°C but not at 42°C (39).

For each positive sample, three *Campylobacter*-like colonies were selected based on their distinct morphological characteristics, including growth speed and grayish or silverish, flat and irregular shapes. These colonies were then purified via two rounds of cultivation on Mueller-Hinton (MH) agar (BD Difco, Fisher Scientific, Toronto, ON, Canada) plates supplemented with 5% defibrinated sheep blood (Alere Inc., Stittsville, ON, Canada). During each round, the plates were cultured at 37°C under microaerobic conditions for 48 h. Single colonies with *Campylobacter*-like morphology were selected for further analysis.

The purified isolates were subjected to species verification using a previously described multiplex PCR (m-PCR) protocol (40), which simultaneously detected *C. jejuni*, *C. coli,* and the *Campylobacter* genus. The primers used for this step are listed in Table S5. Verified *Campylobacter* isolates were preserved in 50% (vol/vol) glycerol and stored at −80°C.

An intuitive naming scheme was applied to all isolates in this study. Each isolate was designated as “(Week ID)-(Sample ID)(Colony ID)”. Week IDs are listed in Table 1 and sample IDs are listed in Table 2. Colony IDs (A/B/C) correspond to the three single colonies selected from each positive sample. For example, the second single colony isolated from the eviscerator swab collected on 9 November 2020 would be named R2S2-8B.

### DNA extraction, library preparation, and sequencing

*Campylobacter* isolates stored at −80°C were revived by cultivating on MH blood agar at 37°C under microaerobic conditions. Following incubation, isolates were selected and verified using m-PCR as aforementioned. Verified *Campylobacter*-positive isolates were then prepared for DNA extraction. Genomic DNA was extracted using the Qiagen DNeasy Blood and Tissue Kit (Qiagen, Toronto, ON, Canada). DNA quantitation was performed using the Quant-It plate assay (Thermo Fisher Scientific, Toronto, ON, Canada). Library preparation and sequencing of the samples were carried out at the Michael Smith Genome Sciences Centre (Vancouver, BC, Canada). Libraries were prepared with a read length of 150 bp, and WGS was performed on an Illumina HiSeq 2000 platform.

### Quality control, assembly, and contamination check

The complete pipeline of quality control and contamination check is depicted in Fig. S2. The base quality of the paired-end sequencing raw files was examined using FastQC (v.0.11.8) (41) with results summarized using MultiQC (v.1.18) (42). Isolates with a sequencing depth less than 10× were excluded from the study. Species abundance in the read files was assessed using Kraken 2 (v.2.1.2) (43). Adapter trimming and removal of low-quality reads were performed using FASTP (v.0.20.0) (44). Bases with a Phred quality score of 15 or lower were neutralized, and reads containing 40% or more neutralized bases were depleted. Genome assembly was performed using the Shovill assembly pipeline (45), specifying SPAdes (46) as the assembler, and the quality of the assembly was assessed using QUAST (v.5.2.0) (47). Genome completeness was evaluated using BUSCO (v.5.4.7) (48). *In silico* MLST was performed following the scheme provided by PubMLST (49). Ribosomal MLST (rMLST) was carried out to determine species information using the rMLST scheme provided by PubMLST (50) via its RESTful API. Isolates containing contaminants detected by Kraken2 or rMLST, as well as those with multiple MLST allelic profiles in more than two loci, were identified as contaminated and removed from the study. A detailed metadata table of all 324 verified isolates is provided in Table S1.

### Characterization of the assembled genomes

Gene prediction and annotation for the assembled genomes were performed using Bakta (v.1.8.1) (51), with *C. jejuni* NCTC 11186 as the reference (52, 53). Genomic features associated with antibiotic resistance were identified using AMRfinderPlus (54). The expression level of *OXA-61*-like genes was predicted by examining the G→T transversion located 57 bp upstream of the start codon using a custom R script (28). Plasmid prediction and typing were performed on the assemblies using MOB-suite (23, 24). Two plasmids were excluded from the MOB-suite plasmid database: plasmid CP023447, due to its high number of chromosomal genes, and plasmid CP013117, due to the presence of a prophage region that could lead to false predictions of prophages as plasmids. Manual curation was conducted to exclude falsely predicted plasmids by examining the gene annotations and assembly graphs for each MOB-suite cluster. Predicted plasmids were excluded if they contained substantial chromosomal sequences or they could not form a closed-loop structure in the assembly graphs. The AMR and plasmid profiles were visualized using the GGTREE package in R (55).

### Core-genome MLST, assignment of lineages, variant calling, and time-based tree construction

Chewiesnake (56), a Snakemake-compiled pipeline (57) based on chewBBACA (58), was applied to generate the cgMLST profiles using the *Campylobacter* cgMLST scheme (59) available on PubMLST. Clustering was performed with a threshold of 20 allelic differences, and a neighbor-joining tree was constructed using GrapeTree (60). Each cluster was assigned as a different lineage. SNP calling was performed to analyze population structures within each lineage. A reference-free approach was applied to the raw reads using DiscoSNP (61), and the identified SNPs and short indels were mapped to the reference sequence *C. jejuni* NCTC 11168. Gubbins (62) was used to determine the presence of homologous recombination regions. A maximum-likelihood SNP tree was generated using RAxML (63), with recombination regions masked. The accessory gene profiles of isolates within each lineage were determined using Roary (64), which used gene annotation outputs from Bakta. To construct a time-based phylogeny, temporal information was integrated into the recombination-removed SNP phylogeny using the BactDating R package (65). The root of the SNP phylogeny was established using sequences from 10 isolates (ST-1629) collected in 2013 in BC as the outgroup (Fig. S3; Table S4). Root-to-tip regression was conducted using the algorithm provided by BactDating, and the time-based phylogeny was constructed using the strict gamma model.

### Detection of subpopulations

For both SNP and accessory gene profiles, linkage disequilibrium was assessed by calculating the pairwise Pearson correlation coefficient (*r*^2^) matrix, using the following equation:


$$r^{2}=\frac{{\left(p_{AB}-p_{A}p_{B}\right)}^{2}}{p_{A}p_{B\left(1-p_{A}\right)\left(1-p_{B}\right)}}$$

where $p_{A}$ and $p_{B}$ represent the allele frequency of features A and B, respectively, and $p_{AB}$ is the allele frequency of both features. When two or more strains share 10 or more highly correlated (*r*^2^ ≥ 0.7) SNP pairs (or three or more highly correlated gene pairs), the $F_{ST}$ of this population is examined for each feature. The $F_{ST}$ for a suspected population at given loci is calculated by the following equation:


$$F_{ST}=\frac{\bar{p}(1-\bar{p})-\bar{p(1-p)}}{\bar{p}(1-\bar{p})}=1-\frac{cp(1-p)+(1-c)p^{\prime }\left(1-p^{\prime }\right)}{\bar{p}(1-\bar{p})}$$

where c represents the ratio of the number of isolates in the suspected population to the total population, p is the allele frequency within the suspected subpopulation, p` is allele frequency in the remaining population (i.e., the population excluding the suspected subpopulation), and $\overset{-}{p}$ is the allele frequency in the whole population. Subpopulations are determined when five or more high-$F_{ST}$ features ($F_{ST}$ > 0.7) exist within a suspected population.

## Data Availability

The raw sequencing reads of the confirmed *Campylobacter* isolates have been submitted to the National Center for Biotechnology Information (NCBI) Sequence Read Archive (SRA) under BioProject accession no. PRJNA930000. The accession numbers for the raw sequence files and genome assemblies of the isolates used in this study can be found in Table S1. For the construction of Fig. 7, 25 publicly available genomes were retrieved from the NCBI Pathogens database (Table S4). As SNP cluster assignments in the NCBI Pathogens database may be subject to reclassification, the SNP cluster names referenced in this study were retrieved on 11 November 2023 (Table 3).

## SUPPLEMENTAL MATERIAL

Supplemental material aem.02369-24-s0001.docx Figures S1 to S3; Table S5.

Table S1 aem.02369-24-s0002.xlsx List of all sequenced isolates in this study.

Table S2 aem.02369-24-s0003.xlsx List of all associated public isolates analyzed in this study.

Table S3 aem.02369-24-s0004.xlsx Summary of all public Washington isolates.

Table S4 aem.02369-24-s0005.xlsx List of all lineage 1629a and 1629b isolates, associated public isolates, and outgroup isolates.

ASM does not own the copyrights to Supplemental Material that may be linked to, or accessed through, an article. The authors have granted ASM a non-exclusive, world-wide license to publish the Supplemental Material files. Please contact the corresponding author directly for reuse.
