# Supplementary material for: Longitudinal and cross-sectional sampling and whole genome sequencing of Campylobacter in a chicken abattoir reveal highly dynamic population structure
Source: Appl Environ Microbiol. 2025 May 9;91(6):e02369-24. doi: 10.1128/aem.02369-24 (PMC12175525; doi:10.1128/aem.02369-24)
Supplement: Supplemental material — Figures S1 to S3; Table S5. [file aem.02369-24-s0001.docx]

**Supplementary Information**

**Longitudinal and Cross-sectional Sampling and Whole Genome Sequencing of *Campylobacter* in a Chicken Abattoir Reveals Highly Dynamic Population Structure**

Shanwei Tong,^abc^ Kaidi Wang,^cd^ Shenmiao Li,^cd^ Michael Trimble,^a^ Yunxuan Chen,^c^ Lixue Liu,^cd^ Jun Duan,^a^ Eduardo Taboada, ^e^ Xiaonan Lu,^cd*^ William Hsiao^a*^

^a^ Center for Infectious Disease Genomics and One Health, Faculty of Health Sciences, Simon Fraser University, Burnaby, British Columbia, Canada

^b^ Bioinformatics Graduate Program, The University of British Columbia, Vancouver, British Columbia, Canada

^c^ Food, Nutrition and Health Program, Faculty of Land and Food Systems, The University of British Columbia, Canada

^d^ Department of Food Science and Agricultural Chemistry, Faculty of Agricultural and Environmental Sciences, McGill University, Ste Anne de Bellevue, Quebec, Canada

^e^ [National Microbiology Laboratory, Public Health Agency of Canada, Winnipeg, Manitoba, Canada](https://www.microbiologyresearch.org/search?option1=pub_affiliation&value1=%22National+Microbiology+Laboratory%2C+Public+Health+Agency+of+Canada%2C+Winnipeg%2C+Manitoba%2C+Canada%22&option912=resultCategory&value912=ResearchPublicationContent)

^*^ Corresponding authors. X.L.: xiaonan.lu@mcgill.ca; W.H.: wwhsiao@sfu.ca


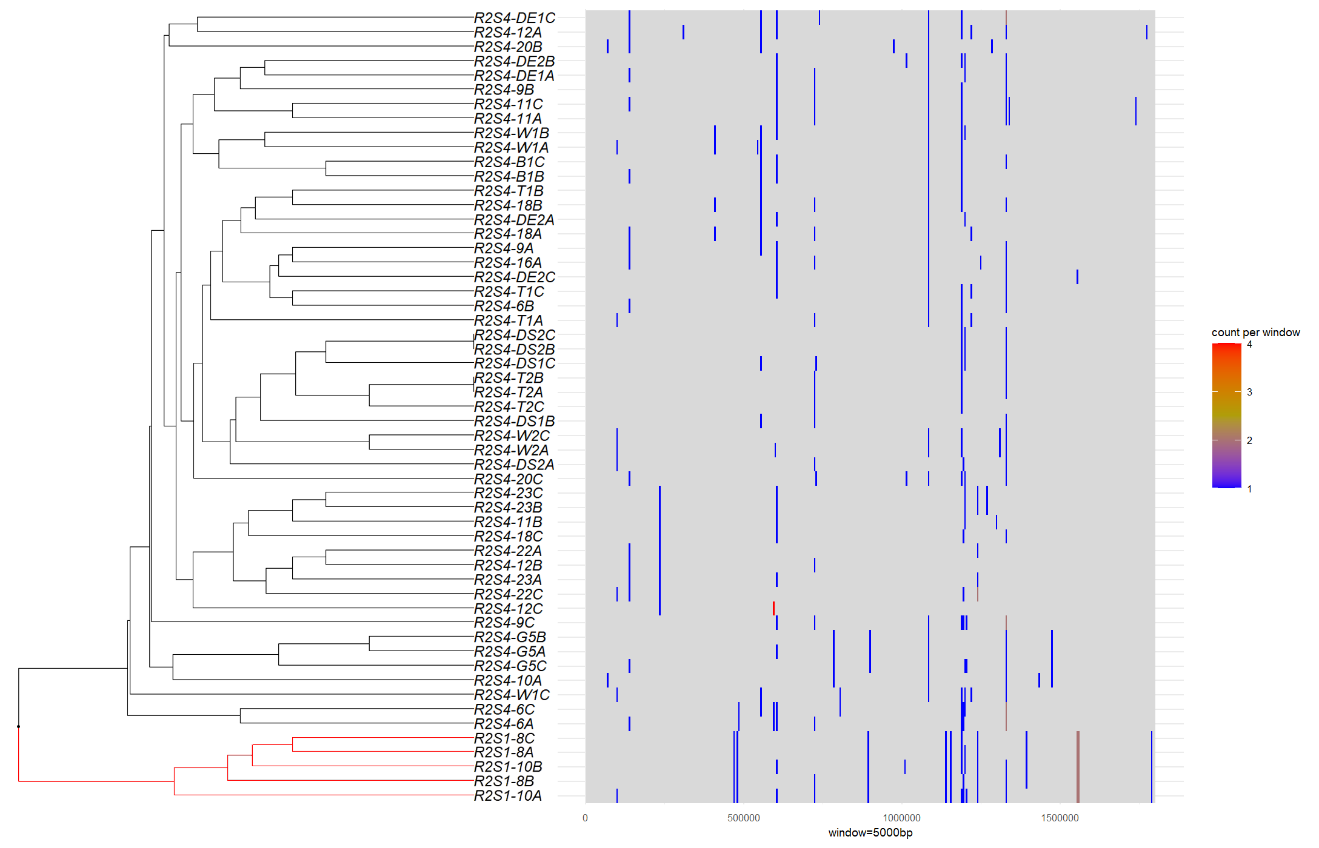


**Figure S1.** Maximum-likelihood SNP tree of lineage 11717 isolates. Point mutations are plotted according to their genomic positions relative to the reference sequence *C. jejuni* NCTC-11168, using a 5,000 bp sliding window. The branch containing week-7 isolates (marked in red) shares a set of 10 point mutations across different regions of the genome.


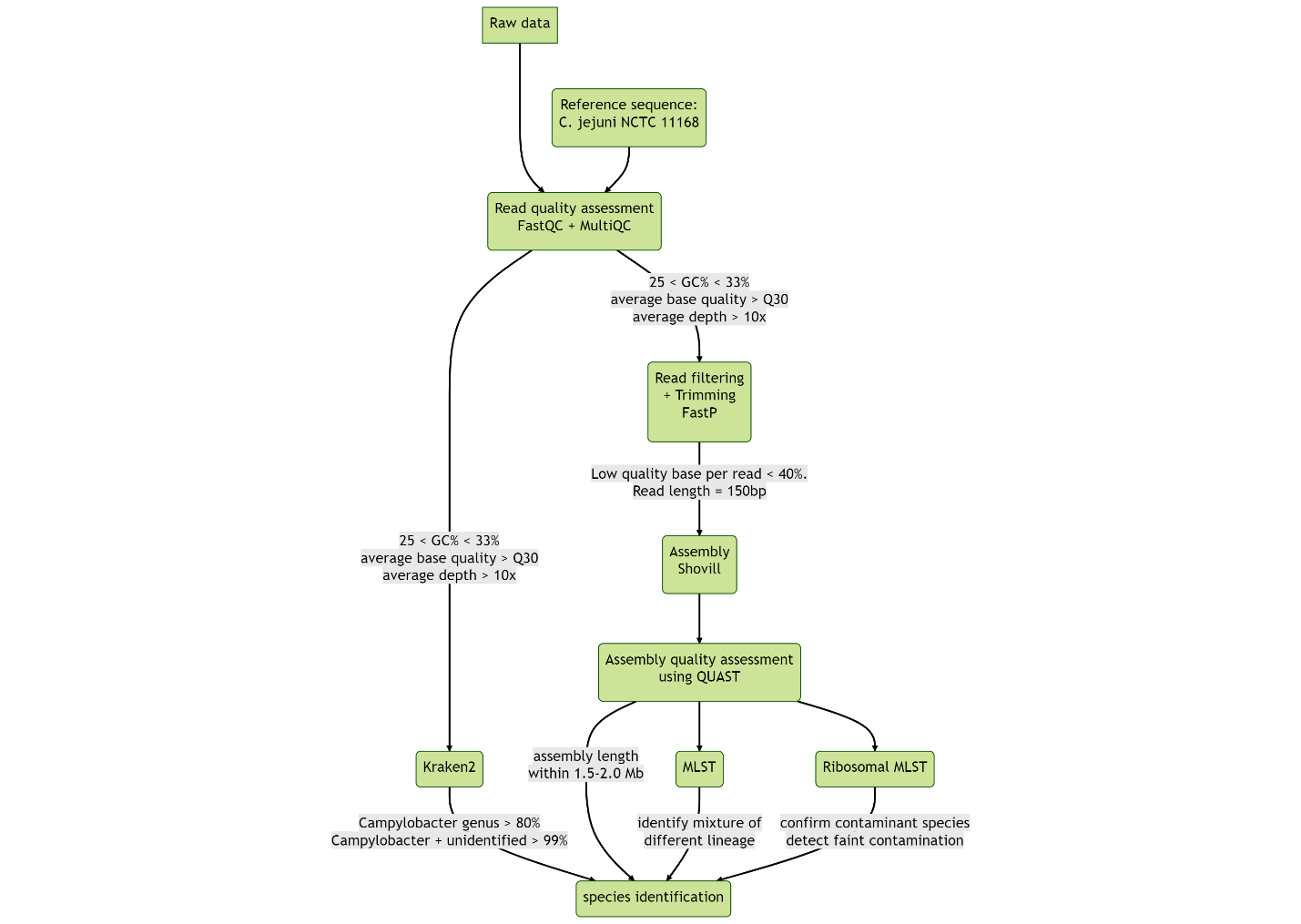


**Figure S2.** Workflow for whole genome sequencing quality assessment and control.


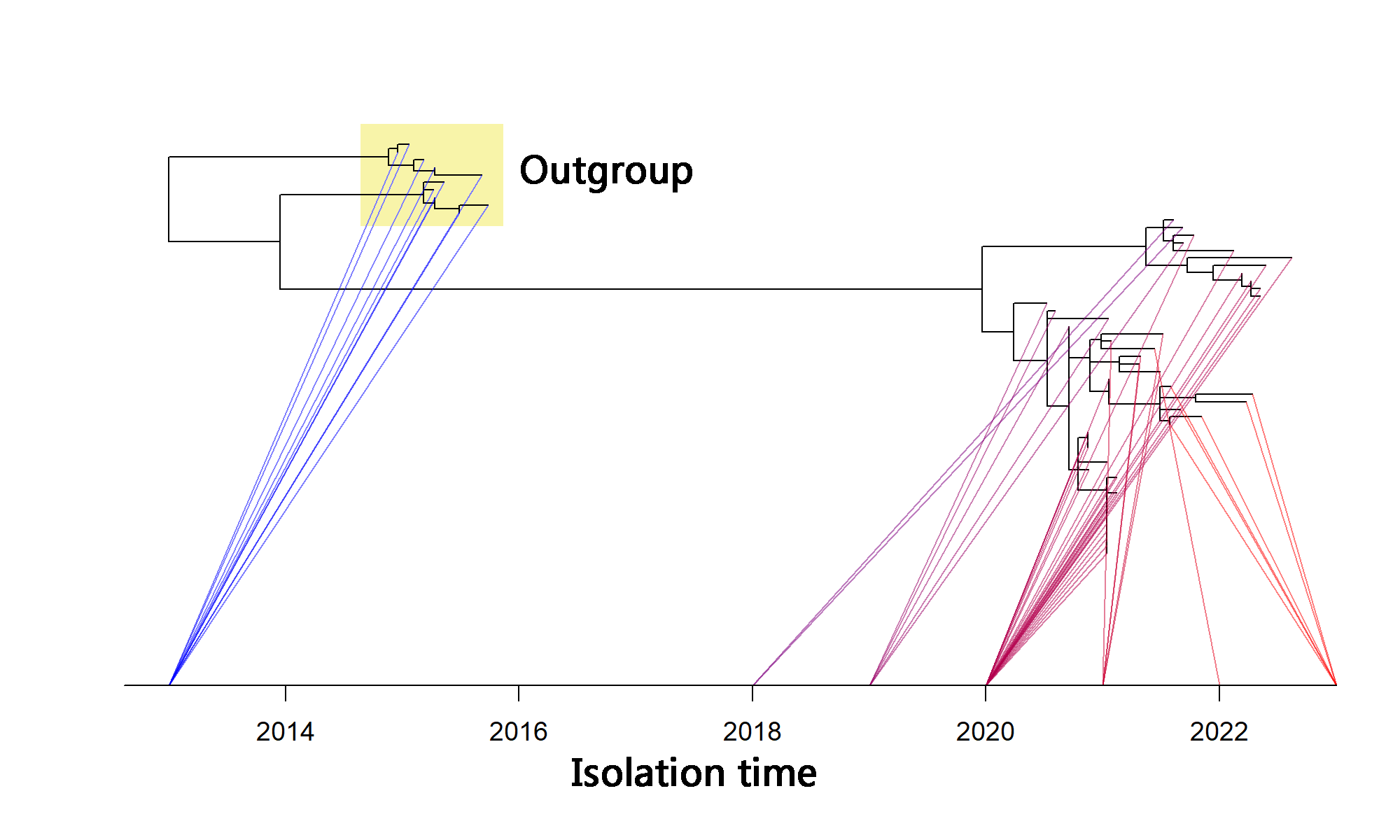


**Figure S3.** Core-genome SNP maximum-likelihood phylogeny of lineage 1629a+1629b isolates along with their associated public isolates (right branch), and 10 outgroup isolates (ST1629) collected from the BC province in 2013 (left branch, highlighted). The node of each isolate is linked to its respective isolation time on the x-axis. The outgroup isolates determined the root position in **Figure 7**.

**Table S1.** List of all sequenced isolates in this study (table_s1.xlsx).

**Table S2**. List of all associated public isolates analyzed in this study. (Attached file table_s2.tsv).

**Table S3**. Summary of all public Washington isolates (table_s3.xlsx).

**Table S4**. List of all lineage 1629a and 1629b isolates, associated public isolates, and outgroup isolates (table_s4.tsv).

**Table S5.** Multiplex PCR primers for the identification of *Campylobacter*

| Primer pair name | Target | Expected band length | Interpretation | Sequence |
| --- | --- | --- | --- | --- |
| Primers A | *mapA* | 589 bp | *C. jejuni* positive | F: ATCTAATGGCTTAACCATTAAAC  R: GGACGGTAACTAGTTTAGTATT |
| Primers B | *ceuE* | 462 bp | *C. coli* positive | F: CTATTTTATTTTTGAGTGCTTGTG  R: CTTTATTTGCCATTTGTTTTATTA |
| Primers C | 16S rRNA | 857 bp | *Campylobacter* positive | F: AATTGAAAATTGCTCCAACTATG  R: TGATTTTATTATTTGTAGCAGCG |
